# Structure-function of proteins interacting with the α_1_ pore-forming subunit of high-voltage-activated calcium channels

**DOI:** 10.3389/fphys.2014.00209

**Published:** 2014-06-03

**Authors:** Alan Neely, Patricia Hidalgo

**Affiliations:** ^1^Centro Interdisciplinario de Neurociencia de Valparaíso and Facultad de Ciencias, Universidad de ValparaísoValparaíso, Chile; ^2^Forschungszentrum Jülich, Institute of Complex Systems 4, Zelluläre BiophysikJülich, Germany

**Keywords:** calcium channels, subunit, ion channels, protein complexes, voltage-dependent channels

## Abstract

Openings of high-voltage-activated (HVA) calcium channels lead to a transient increase in calcium concentration that in turn activate a plethora of cellular functions, including muscle contraction, secretion and gene transcription. To coordinate all these responses calcium channels form supramolecular assemblies containing effectors and regulatory proteins that couple calcium influx to the downstream signal cascades and to feedback elements. According to the original biochemical characterization of skeletal muscle Dihydropyridine receptors, HVA calcium channels are multi-subunit protein complexes consisting of a pore-forming subunit (α_1_) associated with four additional polypeptide chains β, α_2_, δ, and γ, often referred to as accessory subunits. Twenty-five years after the first purification of a high-voltage calcium channel, the concept of a flexible stoichiometry to expand the repertoire of mechanisms that regulate calcium channel influx has emerged. Several other proteins have been identified that associate directly with the α_1_-subunit, including calmodulin and multiple members of the small and large GTPase family. Some of these proteins only interact with a subset of α_1_-subunits and during specific stages of biogenesis. More strikingly, most of the α_1_-subunit interacting proteins, such as the β-subunit and small GTPases, regulate both gating and trafficking through a variety of mechanisms. Modulation of channel activity covers almost all biophysical properties of the channel. Likewise, regulation of the number of channels in the plasma membrane is performed by altering the release of the α_1_-subunit from the endoplasmic reticulum, by reducing its degradation or enhancing its recycling back to the cell surface. In this review, we discuss the structural basis, interplay and functional role of selected proteins that interact with the central pore-forming subunit of HVA calcium channels.

## Introduction

Electrical excitability and synaptic transmission rely on an extended repertoire of voltage-activated ion channels that respond to membrane depolarization by opening an ion-selective pathway across the membrane. Sodium- and potassium-selective channels are mostly involved in the propagation and shaping of electrical signals while calcium channels that are activated during an action potential are responsible for translating changes in the voltage across the membrane into a local calcium increase. This calcium signal initiates a wide spectrum of physiological responses such as muscle contraction, secretion and synaptic transmission (Catterall, 2010). Each one of these processes appears to rely on the formation of multiprotein assemblies with a specialized set of effectors and regulatory proteins that couple calcium influx to downstream signals and feedback controls (Dolphin, 2009; Catterall, 2011). Here, we summarize the structure-function relationship of a set of proteins that interact with and regulate the pore-forming subunit of high-voltage-activated (HVA) calcium channels.

**Figure 1:**
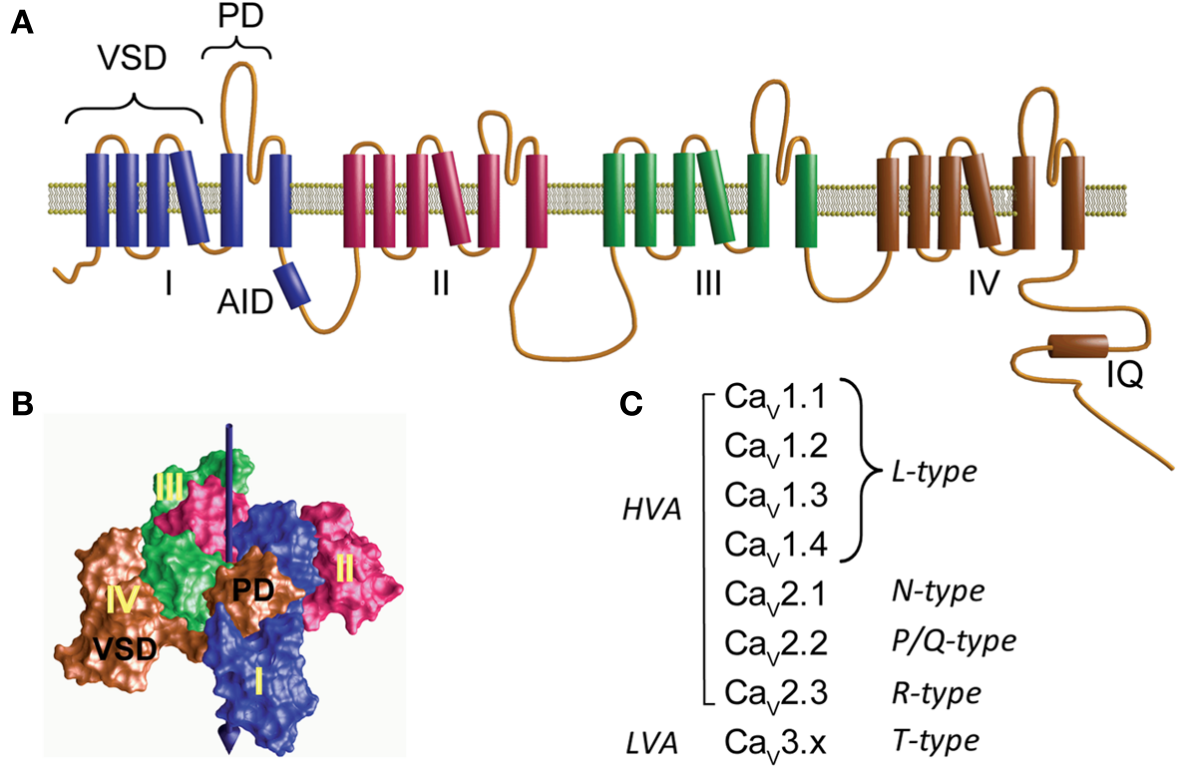
**Structure and diversity of the α_1_ pore-forming subunit of voltage-activated calcium channels**. **(A)** Membrane topology of the α_1_-subunit. Homologous to voltage-activated sodium channel, Ca_V_α_1_ consists of four repeats (I–IV) joined by three cytoplasmic loops namely loop I–II, II–III, and III–IV. Loop I–II contains the highly conserved binding site for the β-subunit, referred to as α_1_ interaction domain or AID. The C-terminus is relatively large and accommodates the binding site for Ca^2+^/calmodulin (IQ). Each repeat contains six transmembrane helices that include two separate structural domains: the voltage-sensing domain (VSD) formed by the first four helices and the pore domain (PD). **(B)** Three-dimensional structure of a tetrameric prokaryotic sodium channel (Na_V_AB, PDB 3VRY) showing four peripheral VDS domains flanking a tetramer of PDs. Note that each VSD flanks the PD domain of a neighboring repeat. The central arrow points to the permeation pathway. **(C)** Ca_V_α_1_ subunits and the corresponding type of Ca^2+^ current that they yield. Ca_V_1.x and Ca_V_2.x correspond to the channel subunit activated by large depolarization (high-voltage-activated, HVA). Ca_V_3.x corresponds to channels activated by low voltages (LVA).

At the heart of the calcium channel protein complex is the α_1_ pore-forming subunit with a design similar to that of the voltage-activated sodium channel consisting of four daisy-chain repeats resembling a subunit of voltage-activated potassium channels (Figure 1A) (Catterall et al., 2005; Catterall, 2011). Each repeat comprises six transmembrane helices flanked by cytoplasmic segments and encompasses two structural domains: the voltage-sensing domain (VSD) formed by the first four helices (S1–S4) and the pore domain (PD) formed by a combination of the last two helices with a membrane-associated loop between segments S5 and S6 (PD, Figure 1A). The VSD and PD from each of the four repeats together form the voltage-sensor and ion-conduction pathway, respectively (Figure 1B). The extracellular aspect of the ion-transport pathway is flanked by the loops joining the S5 and S6 segments while VSDs are located at the periphery (Figure 1B).

The first systematic nomenclature describing the 10 genes encoding for α_1_-subunits used a capital letter referring to the skeletal muscle as α_1S_ and to the rest as α_1A_ through α_1I_ (Birnbaumer et al., 1994). To include the structural relationship between the different α_1_-subunits, a new classification was proposed that resembled the nomenclature used for other members of the voltage-activated ion channel family: Ca_V_x.x; where Ca stands for Ca^2+^, which is the main permeant ion, and V, indicated as a subscript, stands for “voltage,” which is the main physiological regulator (Catterall et al., 2003, 2005). Three structurally and functionally related α_1_-subunit (Ca_V_α_1_) families are distinguished, Ca_V_1.x, Ca_V_2.x, and Ca_V_3.x (Figure 1C). While Ca_V_1.x and Ca_V_2.x correspond to the HVA channels, Ca_V_3.x gives rise to low-voltage-activated channels (LVA) (Ertel et al., 2000).

The α_1_ pore-forming subunit of HVA associates with at least two other subunits: α_2_δ and β(for review see Dolphin, 2012). Each of these subunits is encoded by several genes further diversified by alternative splicing, but for each tissue and cellular context the channel complex appears to be composed of a particular set of subunits. Based on electrophysiological recordings, HVA channels can be separated into four types of currents: L-,N-, P/Q-, or R-type calcium currents. The L-type, so called since it is a long-lasting current in contrast to the transient or T-type currents, is represented by all members of the Ca_V_1.x subfamily while type N, P/Q, and R correspond to individual members of the Ca_V_2.x class (Catterall et al., 2005) (Figure 1C). T-type currents or LVA result from the expression of the Ca_V_3.x subfamily (Figure 1C). LVA channels specialize in shaping the electrical activity of the host cell rather than participating directly in calcium signaling and appear to consist only of an α_1_-subunit and will not be discussed further here (for a review see Perez-Reyes, 2006).

L-type calcium channels correspond to classical dyhidropyridine (DHP) receptors found in the different types of muscle and also in neurons. Hofmann et al. (2014) reviewed very recently in depth L-type calcium channels correlating findings *in vitro* to *in vivo* function. There are four genes coding for the α_1_ pore-forming subunit of L-type channels. Of these, CACNA1C and CACNA1S are the only ones expressed in muscle cells. Neurons and secretory cells express CACNA1C and CACNA1D while CACAN1F appears to be confined to the retina (Baumann et al., 2004). The other types of calcium currents are exclusive of neuronal tissue and they are each represented by a single gene. The N-type current, characterized by being fast inactivating and selectively blocked by Conus toxin GVIA is coded by CACNA1B (Williams et al., 1992a). P-type currents, initially characterized in Purkinge cells (Llinas et al., 1989), were found to be selectively blocked by a toxin isolated from the venom of the funnel web spider *Agelenopsis aperta* (ω-Aga IVA) (Mintz et al., 1992) while Q-type currents originally described in granule cells were eliminated by low concentrations of the Conus toxin MVIIC (Hillyard et al., 1992; Randall and Tsien, 1995), but later heterologous expression experiments showed that the α_1_-subunit encoded by CACNA1A gives rise to both types of currents (Zhang et al., 1993). Finally, the R-type current, so called because it is resistant to the other known calcium channel blockers such as DHP, ω-Aga IVA and Conus toxin GVIA, is encoded by CACNA1E (Schneider et al., 1994; Williams et al., 1994).

## Stoichiometry of high-voltage-activated calcium channels

Initial purification and biochemical characterization of calcium channels from skeletal muscle identified four separate polypeptide chains that co-purified with the DHP receptor. The largest component of 190 kDa corresponds to the α_1_ pore-forming subunit followed by the heavily glycosylated α_2_-subunit of about 170 kDa. Then there is the β-subunit of about 55 kDa that is soluble and intracellular, followed by the membrane-bound components γ-subunit (56 kDa) and δ-subunit (31 kDa) (Leung et al., 1987; Takahashi et al., 1987). The δ-subunit is covalently bound to the α_2_-subunit through disulfide bridges after being translated together in one polypeptide chain and later proteolytically cleaved (De Jongh et al., 1990). The use of the α_1_ and α_2_ nomenclature arose from the initial purification in a non-reducing condition in which α_2_ and δ remained together and migrated to the same position as the pore-forming subunit in SDS electrophoresis (Curtis and Catterall, 1984; Schmid et al., 1986; Leung et al., 1987). To this day it is well accepted that these subunits are stoichiometric components of the calcium channel complex found in vertebrate skeletal muscle and formed by the pore-forming Ca_V_1.1 α1-subunit, α_2_/δ_1c_, β_1a_ and γ_1_ (Dolphin, 2009; Catterall, 2011).

In heart and other tissues, purification of calcium channels has not yet provided a clear-cut answer about the composition and stoichiometry of the channel complex as in skeletal muscle (for a recent review see Hofmann et al., 2014). Several peptides were isolated in the initial purification of DHP receptors from ventricular tissue but only two were recognized as bonafide α_1_ and α_2_δ subunits (Cooper et al., 1987; Chang and Hosey, 1988; Hofmann et al., 1988). Several peptides of smaller molecular mass, ranging from 60 to 25 kDa, were also found (Kuniyasu et al., 1992). It is now well recognized that Ca_v_1.2 (α_1C_) is the main L-type α_1_-subunit responsible for excitation-contraction coupling in the adult heart (Bers, 2002; Larsen et al., 2002). The cloning of β_1a_ from skeletal muscle (Ruth et al., 1989) paved the way for the identification of the heart homolog of this subunit. Several isoforms of the β-subunit were identified in the initial cloning effort (Perez-Reyes et al., 1989; Hullin et al., 1992; Castellano et al., 1993). In other tissues, calcium channels seem more tolerant to the specific subunit that they bind to. With the development of subunit-specific antibodies for the four different isoforms of the β-subunit (β_1_ to β_4_), β_2_ was recognized as the prevalent isoform in the developing and adult murine and human heart (Link et al., 2009). A comprehensive survey by Kamp's groups on human and canine ventricles identified a total of 18 different transcript variants covering all four β genes and displaying differential cellular localization, including T-tubule and surface sarcolemma (Foell et al., 2004). The authors proposed the existence of different populations of Ca_V_1.2 calcium channel complexes with different subunit compositions. Furthermore, subcellular populations of L-type channels with separated functional roles have been suggested (Best and Kamp, 2012). In this context, Ca_V_1.2 complexes localized in T-tubules are mainly involved in excitation-contraction coupling while the complex sited in the plasma membrane outside the invaginations plays a major role in regulating gene transcription.

The contribution of the γ-like subunit to the cardiac calcium channel complex is uncertain. Four isoforms of this subunit are expressed in the human heart and all associate with heterologously expressed cardiac calcium channels and modulate function to varying degrees (Yang et al., 2011). The α_2_δ subunit isolated from the heart cross-reacts with antibodies raised against its homologs from skeletal muscle (Cooper et al., 1987; Chang and Hosey, 1988) and following its cloning it was confirmed to be encoded by the same gene (Ellis et al., 1988).

The scenario is still more complex in the neuronal tissue where all Ca_V_1.x, except the skeletal muscle isoform (Ca_V_1.1), are found. Identifying the different kinds of calcium channel complexes and determining their exact subunit composition was a rather daunting task. They all appear to share the basic combination of an α_1_ pore-forming subunit associated with one α_2_δ- and one β-subunit while interaction with a γ-subunit is less certain. However, calcium channels are increasingly viewed as multi-protein assemblies that compose signaling complexes. For example, in the heart calcium channel supramolecular complexes might involve protein phosphatases, G proteins, adenylcyclases, A-kinase anchoring proteins, phosphodiesterases, protein kinases and β_2_ adrenergic receptors (Harvey and Hell, 2013). Here we revise the structure-function of proteins that co-purified with the DHP receptor from skeletal muscle: α_2_δ, β, and γ (Leung et al., 1987; Takahashi et al., 1987). We also include calmodulin (CaM), believed to be a rather stable partner of the α_1_ pore-forming subunit, and small GTPase for their importance in channel regulation (Minor and Findeisen, 2010). There are many other proteins, such the βγ complex of receptor-regulated G-protein, HSP70, synaptotagmin, and cysteine string proteins, that have been identified in calcium channel complexes which we omitted and which are reviewed elsewhere (Kobayashi et al., 2007).

## Alpha2-delta subunit

The largest proteins partner of α_1_ is the α_2_δ-subunit. It is a membrane protein with molecular masses between 140 and 170 kDa that is split into two moieties by post-translational proteolysis (α_2_ and δ), which are held together through disulfide bonds (De Jongh et al., 1990). Two mechanisms have been postulated to explain membrane anchoring of the α_2_δ-subunit: the δ polypeptide chain either forms a transmembrane α-helix (Calderon-Rivera et al., 2012) or it undergoes a post-translational modification that adds a lipid binding moiety to the peptide (Davies et al., 2010). The functional role of the α_2_δ-subunit is mostly inferred from electrophysiological studies co-expressing α_2_δ with different α_1_ pore-forming subunits in heterologous expression systems. The α_2_δ-subunit enhances current densities, speeds up activation and inactivation kinetics and produces a hyperpolarizing shift in the voltage dependence of inactivation (Singer et al., 1991; Williams et al., 1992b).

**Figure 2:**
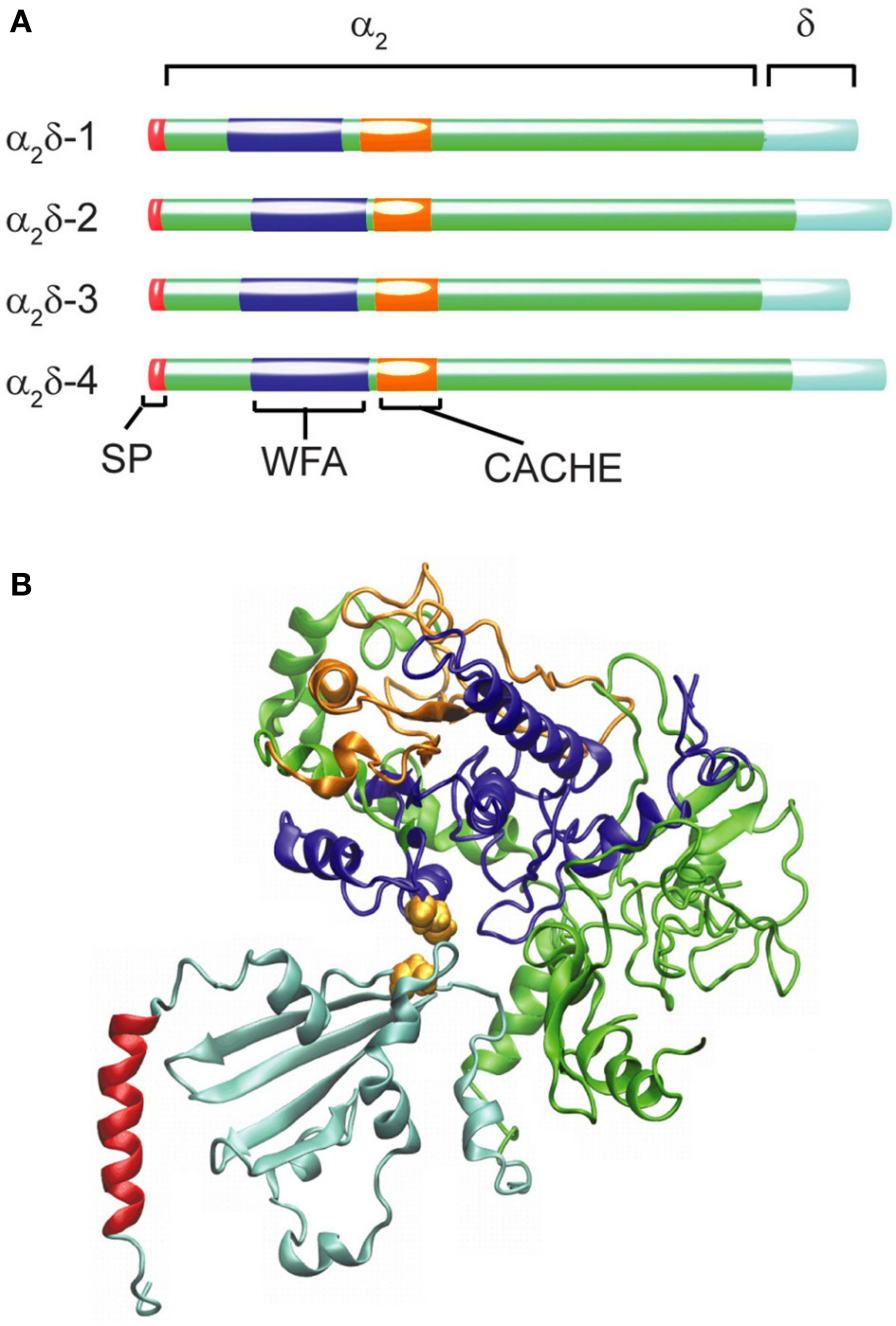
**Structural organization and diversity of calcium channel α_2_δ subunits**. **(A)** Schematic representation of the domain arrangement of the four α_2_δ isoforms identified in humans scaled according to the number of amino acids (α_2_δ-1 acc. ·P54289; α_2_δ-2 acc. Q9NY47; α_2_δ-3 acc. Q8IZS8; α_2_δ-4 acc. Q7Z3S7). The N-terminal signal peptide (SP) ranging from 19 amino acids for (α_2_δ-2) to 29 residues for α_2_δ-3 shown in red is not present in the mature protein. The blue segment depicts the WFA domain and the orange one the CACHE domain. **(B)** Ribbon diagram of the model proposed by Calderon-Rivera et al. (2012) showing the WFA domain in blue and CACHE in orange while the rest of the α_2_ peptide is depicted in green. Within the δ peptide (cyan) an alpha helical segment (shown in red) is likely to represent a membrane-anchoring domain. The cysteine residues required for the α_2_δ assembly are represented as van der Waals surfaces in yellow.

In the human genome, four genes code for this subunit, including CACNA2D1, which gives rise to the isoform α_2_δ-1 found in all muscle types as well as in the brain (Figure 2A). The other three genes are expressed in specialized regions of the nervous system: α_2_δ-2 encoded by CACNA2D2 is particularly abundant in the cerebellum, α_2_δ-3 (CACNA2D3) is found in the peripheral nervous system besides the central nervous system and α_2_δ-4 (CACNA2D4) is present in neuroendocrine tissue and in the retina. All these genes give rise to multiple transcripts as the result of alternative splicing (Dolphin, 2013).

The α_2_δ subunit appears to be the only glycosylated proteins that co-purified with the skeletal muscle DHP receptor (Takahashi et al., 1987). The initial cloning of the α_2_δ-encoding mRNA from skeletal muscle (Ellis et al., 1988) revealed that the N-terminus sequence of the mature protein was preceded by a signal peptide 26 amino acids in length (SP, Figure 2A) that is consistent with the extracellular destination of the following segment of about 900 residues containing many potential N-glycosylation sites (Nakayama et al., 1987). Sequencing of tryptic fragments of the δ-subunit purified from skeletal muscle matched with sequences upstream of residue 900 from the cloned sequence proving that α_2_ and δ were synthesized as a single peptide chain and identified the cleavage site between Glu934 and Ala935 (De Jongh et al., 1990).

Additional insight into the architecture of the extracellular α_2_ peptide comes from bioinformatic analysis that identified the presence of a von Willebrand factor A domain (WFA, Figure 2A). The WFA domain, about 200 amino acids in length, binds to a variety of cell adhesion extracellular matrix proteins (Whittaker and Hynes, 2002). It contains two metal-ion-dependent adhesion sites (MIDAS) that participate in protein-protein interactions and that are required to promote the anterograde transport of Ca_V_α_1_ (Canti et al., 2005; Hoppa et al., 2012). At a short distance downstream of the WFA domain, a bacterial chemosensory-like domain (CACHE, Figure 2A) can be recognized. CACHE is a signaling domain shared by bacterial chemotaxis receptors and calcium channel α_2_δ-subunits in animals (Anantharaman and Aravind, 2000). Taking advantage of these conserved domains with known crystal structures, a model of the three-dimensional structure of the α_2_δ-subunit was generated and structure-function correlation studies defined a particularly relevant intermolecular disulfide bond (Calderon-Rivera et al., 2012) (Figure 2B, see also below).

The most widely observed consequence of co-expression of α_2_δ with any α_1_/β channel combination is an increase in current density that correlates with a larger number of ligand-binding sites for the channel (Felix, 1999; Arikkath and Campbell, 2003; Klugbauer et al., 2003). This observation leads to the broadly accepted view that α_2_δ is involved in the regulation of traffic and stability of the channel protein complex. Besides this effect on the number of surface channels, the presence of this subunit has been correlated with a shift in the voltage dependence of activation toward more negative voltages and an increase in the rate of voltage-dependent activation and inactivation. The magnitude and relative importance of these effects depend on the expression system and the particular subunit combination being tested. For example, Ca_V_2.3 expressed in *Xenopus* oocytes did not display an increase in current density with α_2_δ-1 (Jones et al., 1998). There are other reports on α_2_δ showing no effect on the voltage dependence of activation (Singer et al., 1991; Shistik et al., 1995; Bangalore et al., 1996), but in all these studies a unique palmitoylable β-subunit, β_2a_ (Chien et al., 1996; Qin et al., 1998), was used. In contrast, when co-expressing Ca_V_1.2 with β_3_, the addition of α_2_δ shifted the current-voltage relationship by about −10 mV. However, a more dramatic effect of α_2_δ was observed on the pre-pulse facilitation of the channel than in the voltage-dependent activation curve (Platano et al., 2000). Pre-pulse facilitation refers to the transient increase in calcium currents that follows a prolonged depolarization (pre-pulse) to extreme positive voltages (Dolphin, 1996) and that depends on the subunit composition of the channel complex (Bourinet et al., 1994; Cens et al., 1996, 1998; Qin et al., 1998; Dai et al., 1999). The voltage-dependence of activation of Ca_V_1.2/β_3_/α_2_δ channels nearly follows that of Ca_V_1.2/β_3_ channels after pre-pulse facilitation as if calcium channels were permanently facilitated by α_2_δ, indicating a functional overlap between α_2_δ and β.

Proteolytic processing is a rather unique feature of α_2_δ among VWA-containing proteins (Whittaker and Hynes, 2002) shared only by CCLA proteins that were originally thought to constitute calcium-dependent chloride conductance but are now viewed as modulators of chloride channels (Loewen et al., 2004; Loewen and Forsyth, 2005). Mutagenesis of the putative proteolytic cleavage site between α_2_ and δ in the neuronal homolog α_2_δ-1b prevented proteolysis in heterologous expression systems such as HEK-293 cells and the increase in the current density of co-expressed Ca_V_2.2/β_3_/mutant α_2_δ channels (Andrade et al., 2007). However, all proteolysis-resistant mutants were capable of accelerating the activation and inactivation kinetics showing that non-cleaved proteins retained their capacity to interact with the mature membrane-localized pore-forming subunit. It may be asked whether controlled proteolysis of α_2_δ may be an additional knob to fine tune calcium currents since it has been shown that there is incomplete proteolysis in several commonly used expression systems, but that in a native preparations, such as cerebellum, only the cleaved form is found (Douglas et al., 2006). An inspiring case is that of the activation of epithelial sodium channels by proteolysis (Svenningsen et al., 2013), although the prevalent view is that proteolysis of α_2_δ occurs while in the ER and that it follows disulfide bond linkage (Dolphin, 2013). Of the six conserved cysteine residues found in the extracellular domain of the δ, only one is critical for function and for holding α_2_ and δ together in reducing conditions (Calderon-Rivera et al., 2012). The partner for this cysteine was identified within the α_2_ moiety. With respect to impaired proteolysis, eliminating either of the two cysteines abolished the increase in current density induced by co-expressing α_2_δ while sparing modulation of activation and deactivation kinetics.

Extensive glycosylation is also a salient feature of the α_2_δ-subunit of calcium channels. To estimate the amount of glycosylation, α_2_δ fragments were translated *in vitro* in the presence and absence of microsomal membranes and their molecular weight compared: The result was 28 kDa of carbohydrates. The functional relevance of these carbohydrates became obvious when treating oocytes expressing Ca_V_2.2/β_4_/α_2_δ complexes with N-glycosidase and finding that current densities returned to the levels observed without α_2_δ(Gurnett et al., 1996). Sequence analysis identified 10 putative N-linked glycosylation sites, two of which were located within the N-terminal segment and were shown by Gurnett et al. (1996) to be necessary for increasing currents. Sandoval et al. (2004) destroyed both sites by replacing the asparagines by glutamines and found that co-expression of the double mutant with Ca2.2/β_3_ channels yielded current densities comparable to those measured in the absence of α_2_δ. Peak currents obtained with each single mutant were similar and halfway between the double mutant and wild type α_2_δ. As observed for the mutation preventing proteolysis and disulfide bond formation, the effects on activation and deactivation were spared. While mutagenesis experiments are consistent with a role of glycosylation in the forward traffic of the channel, the extracellular treatment with glycosidases reduced currents, suggesting an effect on stability and backward traffic instead.

Another functionally relevant post-translational modification of α_2_δ-1-3 is the attachment of a glycosyl-phosphatidylinositol (GPI) moiety to the δ peptide. This is required for increasing the current density of the Ca_V_2.2/β_1b_ channel subunit combination and was proposed to be the membrane-anchoring motif (Davies et al., 2010). However, replacing this motif by a transmembrane spanning helix from an unrelated protein restores membrane anchoring (Robinson et al., 2011). This observation is in line with the membrane topology prediction methods employed by Calderon-Rivera et al. (2012).

Important insight into the physiological role of this auxiliary subunit has been gained by animal models carrying spontaneous mutation-targeted disruption of α_2_δ-encoding genes. Two spontaneous mutations that cause epilepsy and ataxia were mapped to the CACNA2D2 gene and correlated with a reduction of calcium currents recorded from Purkinje cells (Barclay et al., 2001; Brodbeck et al., 2002; Brill et al., 2004). More recently, an inherited form of epileptic encephalopathy was linked to a missense mutation in the CACNA2D2 gene that impairs the ability of this subunit to boost heterologous expression of Ca_V_2.2 and Ca_V_1.2 channels (Edvardson et al., 2013). These findings fit nicely with previous studies showing that the GABA-like antiepileptic drugs Gabapentin and Pregabalin bind α_2_δ-1 and α_2_δ-2 (Gee et al., 1996; Wang et al., 1999; Bian et al., 2006; Field et al., 2006). Although some reports indicate that these drugs produce acute inhibition of calcium currents (Stefani et al., 1998; Martin et al., 2002), the prevailing view is that the pharmacological action involves inhibition of the forward trafficking of Ca_V_2.1 and Ca_V_2.2 following chronic exposure (Kang et al., 2002; Vega-Hernandez and Felix, 2002; Hendrich et al., 2008). Binding of Gabapentin to α_2_δ appears to disrupt Rab11-dependent recycling from late endosomes, preventing the channel complex from returning to the plasma membrane (Tran-Van-Minh and Dolphin, 2010). A similar conclusion emerges from the finding that a mutant prion protein that suppresses glutamatergic neurotransmission does so by binding to α_2_δ-1 and impairing delivery of the channel complex to the pre-synaptic membrane (Senatore et al., 2012). Besides regulating the traffic of the channel, α_2_δ-1 may also play a role in synaptic formation by binding to the synaptogenic traffic factors thrombospondin 1, 2, or 4 (Eroglu et al., 2009). This interaction is mediated by the VWF domain that also mediates protein-protein interactions with integrins and other cell adhesion proteins (Whittaker and Hynes, 2002). Thus, as discussed for the β-subunit (see below), the α_2_δ-2 subunit appears capable of associating with other cellular partners and of fulfilling functions that might not necessarily correlate with the regulation of calcium channel function.

## Beta subunit

**Figure 3:**
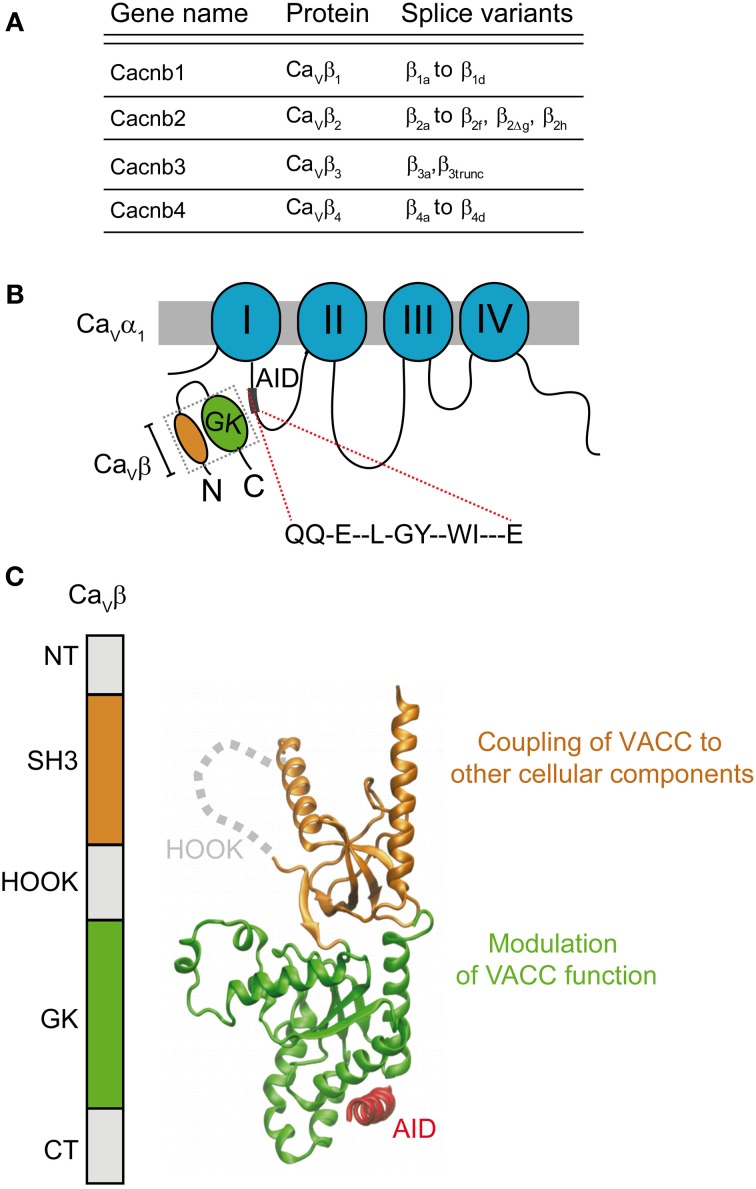
**Gene family and structure of the β-subunit**. **(A)** List of β-subunit encoding genes with splice variants (reviewed by Buraei and Yang, 2010). **(B)** Scheme of the α_1_- and β-subunits showing the consensus amino acid sequence α_1_-interaction domain or AID located within the intracellular I–II loop that binds to the β-subunit guanylate kinase domain (GK). **(C)** Domain organization and three-dimensional structure (PDB 1T3L) of the β-subunit. The SH3 domain is shown in orange and the GK domain in green. The AID peptide forms an alpha helix (shown in red) upon binding to the GK domain. The GK domain is responsible for modulation of function of HVA channels while SH3 appears to couple the channel to other cellular components.

The sine qua non for the normal reconstitution of functional HVA channels in mammalian cells is the presence of the β-subunit. Since its identification as a subunit co-purifying with the DHP receptor from skeletal muscle (Takahashi et al., 1987) and its cloning (Ruth et al., 1989; Lacerda et al., 1991), a whole body of evidence emphasizes the role of the β-subunit in modulating calcium channels, i.e. to increase current density. Four different genes encode four different isoforms (β_1_–β_4_) with several splice variants (for an extensive review see Buraei and Yang, 2010) (Figure 3A). It is clear now that all classical β-subunits upregulate calcium currents using two separate modes: they increase the probability of the channel being in the open state and also of being in the plasma membrane. The β-subunit binds to the α_1_ pore-forming subunit through a main anchoring site shared by all α_1_-subunits belonging to HVA family, referred to as the α_1_ interaction domain or AID (Pragnell et al., 1994). AID is a consensus sequence consisting of 18 amino acids located in the intracellular loop joining repeats I and II of HVA (Figure 3B). Disruption of this site completely eliminates channel modulation, which is fulfilled by the binding of one β-molecule to one α_1_-subunit (Dalton et al., 2005). Physical interaction with other regions of the α_1_-subunit has been reported but the functional role of these contact points is unclear (Maltez et al., 2005; Dolphin, 2012). They may constitute secondary sites that provide extra regulatory points in an isoform-specific manner.

In, 2004, three groups published the crystal structure of three β-subunit isoforms (β_2_, β_3_, and β_4_) alone and in complex with the AID consensus sequence (Chen et al., 2004; Opatowsky et al., 2004b; Van et al., 2004). These studies revealed that the two highly conserved domains of the β-subunit, Src Homology 3 domain (SH3) and guanylate kinase (GK), are structurally related to members of the membrane-associated guanylate kinase (MAGUK) family of proteins (Opatowsky et al., 2004a) (Figure 3C). All β-subunit variants, except for the short splice forms β_4c_ (Hibino et al., 2003) and β_1d_, (Cohen et al., 2005) and a few others found in the heart (Foell et al., 2004), share this architecture (Figure 3). Association of the β-subunit with the AID site induces a coil-to-helix transition in the AID region that likely extends to the S6 segment of the first repeat (Findeisen and Minor, 2009; Gonzalez-Gutierrez et al., 2010). Although a clear mechanism of how the β-subunit facilitates pore opening or forward trafficking of the channel was not readily deducible from the three-dimensional structures, these studies opened up new scenarios to explain β-modulation. Only one module, the GK, binds to the AID and their interaction surface lies opposite to the SH3 domain (Figure 3C) suggesting that GK might suffice for channel modulation. Although the SH3-GK core is widely viewed as the minimal unit for channel modulation including trafficking (Takahashi et al., 2004, 2005; He et al., 2007), in our work we found that recombinant GK was sufficient to restore normal channel gating of Ca_V_2.3 channels expressed in *Xenopus* oocytes (Gonzalez-Gutierrez et al., 2008b).

The increase in current density induced by the β-subunit is accompanied by a shift in the activation threshold to more negative voltages. Simultaneous recording of gating currents, which report the movement of the channels' voltage sensor, and ionic currents showed that a relevant fraction of the current density increase was due to an augmented current-carrying capacity of the channel (Neely et al., 1993). By comparing the effect of the β_2a_ on the activation of Ca_V_1.2, and Ca_V_2.3, the concept emerges that the β-subunit decreases the charge to ionic current ratio or increases the coupling efficiency of the voltage sensing structures to the pore (Olcese et al., 1996). Single-channel studies showed that the probability of the channel being open is increased (Costantin et al., 1998). A salient feature of HVA channels is that single channels wander between different gating modes complicating the analysis and identification of the particular steps being regulated by the β-subunit (Dzhura and Neely, 2003; Luvisetto et al., 2004; Jangsangthong et al., 2011). Certainly, all β-subunits increase the electrical activity of the channel, but it is not clear why the effect on the voltage dependence of the activation is more pronounced for Ca_V_1.2 than for Ca_V_2.3 channels. For example, in the *Xenopus* oocyte expression system, β_2a_ left-shifts the activation curve about 60 mV compared with the channel alone and a shift of around 8 mV is observed for Ca_V_2.3 (Olcese et al., 1996). Since cytoplasmic loops start as an extension of the sixth segment of each repeat, they are likely to influence channel gating. We swapped the I–II loop of the poorly coupled Ca_V_1.2 channel with the efficiently coupled Ca_V_2.3 and found that these phenotypes were transferred independently of β-subunit interaction (Gonzalez-Gutierrez et al., 2010). When the β-subunit is present, the coupling efficiency is sensitive to the residues within the AID domain that are exposed to the milieu (Gonzalez-Gutierrez et al., 2008a). Taking these results together, it appears that structural determinants within the I–II loop interact with the channel gating machinery and that binding to the β-subunit modulates this interaction by repositioning yet to be identified elements. However, this is still an imprecise picture of the mechanisms underlying regulation of voltage-sensing to channel-opening coupling by the β-subunit. An important obstacle to resolve this issue is that expression of functional channels in the absence of β only appears possible in *Xenopus* oocytes and it is so far restricted to rabbit Ca_V_1.2 and human Ca_V_2.3. The ability of the β-subunit to increase the open probability of the neuronal Ca_V_2.1 and Ca_V_2.2 channels remains to be determined.

Voltage-dependent inactivation (VDI) is also subject to modification by the β-subunit in a manner that depends on the particular isoform that associates with the channel. Classic work on Ca_V_2.3 channels showed that most β-subunits accelerate VDI and shift the voltage dependence toward more negative potentials. The only exception was the β_2a_-subunit (Olcese et al., 1994) that was later shown to owe its phenotype to a palmitoylable membrane-anchoring domain (Qin et al., 1998). Recently, we identified another β_2_ isoform, β_2e_, that also inhibits VDI of Ca_V_2.3 channels and whose VDI-inhibiting phenotype depends on a positively charged anchoring membrane domain within the N-terminus (Miranda-Laferte et al., 2014). However, work with purified recombinant β-subunits clearly showed that membrane anchoring is not the only way of inhibiting VDI (Gonzalez-Gutierrez et al., 2008b). For example, GK-Ca_V_2.3 interaction was by itself capable of inhibiting inactivation although GK is not tethered to the plasma membrane by itself. In fact, we found that the β_1b_ isoform which is known to facilitate VDI in coexpression experiments also inhibited VDI when injected as a recombinant protein into oocytes already expressing Ca_V_2.3 in the plasma membrane (Gonzalez-Gutierrez et al., 2008b). If the purified β_1b_ is injected at an earlier time when mature channels are not yet assembled into the plasma membrane, it recovered its canonical fast-conferring VDI phenotype. The role of potential post-translational modifications in the full development of VDI regulatory capabilities of the β-subunit still remains puzzling. In addition, membrane anchoring not only contributes to immobilizing a putative inactivation particle (Stotz et al., 2004) but also exposes another structural element, namely, a polybasic segment identified in the linker joining GK and SH3 that is unique to β_2_-subunits (Miranda-Laferte et al., 2011).

While the molecular mechanisms by which the β-subunit controls channel gating are not fully understood, those underlying regulation of the intracellular forward trafficking are as yet even less clear. The first hypothesis came from experiments showing that the transfer of the I–II loop of Ca_V_2.1 to the Shaker K^+^ channel conferred β-dependent surface expression in *Xenopus* oocytes (Bichet et al., 2000). It was proposed at the time that binding of the β-subunit occludes an endoplasmic reticulum (ER) retention signal located close to the AID site within the I–II loop. In this context, binding of β to channels retained in the ER allows their release and transport to the plasma membrane. More than a decade later, by using a systematic chimeric approach between Ca_V_1.2 and a Ca_V_3.1 that does not exhibit β-subunit affinity, Colecraft's group showed that while the I–II loop increases channel surface density, the rest of the loops including the N- and C-termini have the opposite effect (Fang and Colecraft, 2011). The authors put forward the idea that the I–II loop contains an ER export signal rather than a retention signal that it is silenced by retention signals somewhere within the other cytoplasmic regions. β-association with the AID site would induce structural rearrangements involving the C-terminus that may suppress retention signals and unmask the loop I–II export signal. Irrespective of the model, it appears that association of the β-subunit with immature channels likely sited in the ER is absolutely necessary for normal surface expression of the mature channel, but whether the β-subunit dissociates on its way to the plasma membrane is unclear. The dynamic nature of α_1_−β association is likely determined by the β-subunit subtype, ranging from a rather stable complex for either Ca_V_1.1 or Ca_V_1.2 with β_1a_ to a more dynamic one with β_2a_ and β_4b_ as judged by fluorescence recovery after photobleaching of α_1_ and β-subunits fused to GFP (Campiglio et al., 2013).

The binding affinity for the β/AID interaction has been well studied *in vitro* but assessment of this association *in vivo* appears quite difficult, most likely because it depends on the cellular context where different protein partners might be encountered. The K_D_ values for the α_1_/β interaction obtained with the full pore-forming subunit are significantly larger than that estimated *in vitro* with AID-derived peptides. The K_D_ measured from the effect on channels expressed in *Xenopus* oocytes ranges from 120 nM [Ca_V_2.2 and β_3_ (Canti et al., 2001)] to 350 nM [Ca_V_2.3 and β_2a_ (Hidalgo et al., 2006)], with an intermediate value of 200 nM for Ca_V_1.2 and β_2a_ (Gonzalez-Gutierrez et al., 2008a). Isothermal titration calorimetry studies resulted in K_D_ of 3.5 to 53.5 nM between the functional core derived from β_2a_ (Van et al., 2004) and AID peptides from different HVA α_1_-subunits (Van et al., 2008). Up to now we can merely speculate on what might weaken the association of the β-subunit with channels in intact cells. One possibility is that the other cytoplasmic domains, including the N- and C-termini, reduce the affinity to the main anchoring site simply by steric hindrance. Alternatively, the cytoplasmic regions that provide several binding sites for other regulatory proteins may promote dissociation of the β-subunit in the occupied state.

Enhancement of channel density in the plasma membrane by the β-subunit is not only achieved by promoting the anterograde trafficking but also by preventing proteosomal degradation of Ca_V_1.2 channels (Altier et al., 2011). On the other hand, the β-subunit also impacts the backward trafficking of the channel through its SH3 domain that associates directly with the dynamin GTPase, a protein involved in vesicle fission and promotes endocytosis (Gonzalez-Gutierrez et al., 2007). These findings question the canonical view of the β-subunit as being mostly a positive regulators of calcium currents and opens the possibility that it may switch from being a calcium current activator to be an inhibitor. We postulated that the endocytic effect is regulated by the oligomerization state of this subunit (Miranda-Laferte et al., 2011). Dissociation of the β-subunit would allow the formation of a dimer and its engagement in the endocytic pathway. Soldatov's group also showed higher-order oligomers for several β-isoforms in smooth muscle cells and heterologous COS cell expression systems, but the oligomers were correlated with an increase in calcium current density (Lao et al., 2010). Despite this discrepancy, the quaternary structure of the β-subunit emerges as a novel regulatory input for β-modulation of calcium currents.

The functional flexibility of the β-subunit is expanded not only by its oligomeric state but also by its cellular location. Most of the isoforms are cytosolic except for two splice variants belonging to type 2, β_2a_ and β_2b_. The mechanism of membrane anchoring differs, while for β_2a_ it is the covalent binding of a lipid group (palmitoyl), for β_2e_ an electrostatic mechanism has been proposed (Miranda-Laferte et al., 2014). As described above, membrane anchoring appears to confer to this subunit the unique capability to inhibit VDI. Besides this electrophysiological phenotype, membrane association may divert channels to different compartments during trafficking and assembly. Associations with multiple partners may also broaden the regulatory repertoire. For example, binding to Kir/Gem small GTPases goes as far as translocating the β-subunit to the nucleus (Mahalakshmi et al., 2007a).

The significant effect of the β-subunit on Ca_V_1.2; the most prominent calcium current in the heart, predicts that dysfunction of this subunit results in pathological conditions by altering calcium homoestasis. In mice, knockout of the gene encoding for the predominant β-subunit in the heart, β_2_ (Haase et al., 1996; Chu et al., 2004; Foell et al., 2004; Link et al., 2009) results in heart malformation and embryonic death (Weissgerber et al., 2006) while unexpectedly, the conditional knock-out had only a moderate effect on adults (Meissner et al., 2011). In zebrafish, depletion of β_2.1_ (homologous to human β_2e_) at the embryonic stage also results in ventricular malformations and death (Chernyavskaya et al., 2012). In humans, increased β_2_ expression has been observed in hypertrophic obstructive cardiomyopathy (Haase et al., 1996) and failing heart (Hullin et al., 2007). Why adult mice tolerate β_2_ depletion is unclear (Meissner et al., 2011). It is possible that in the adult heart, Ca_V_1.2 is less sensitive to the β_2_-subunit than as observed in reconstitution or heterologous expression systems. It is believed that in the heart, calcium channels form large multi-molecular signaling complexes (Dai et al., 2009; Harvey and Hell, 2013). The existence of compensatory mechanisms for the calcium influx deficit through these channels, which are independent of the presence of the β-subunit within the supramolecular complex, may explain the flexible requirement of β-subunit expression in adult mice.

It is certain that the β-subunit is much more than a stoichiometry subunit as believed during the first calcium channel purification more than 27 years ago (Jones, 2002). Dynamin is just one of many other partners found in the past decade together with small GTPases (Beguin et al., 2001), chromatin binding protein (Hibino et al., 2003), AHNAK (Hohaus et al., 2002). How these multiple interactions are coordinated within the cell and what role is played by subcellular localization and gene expression remains to be investigated.

## Gama subunit

The initial purification of DHP receptors from skeletal muscle identified a fourth subunit of about 30 kDa as part of the channel complex (Takahashi et al., 1987). A protein of similar molecular mass was not found in preparations from cardiac (Cooper et al., 1987) nor from neuronal tissue (Ahlijanian et al., 1990; Sakurai et al., 1996), suggesting that this subunit, γ_1_, was unique to skeletal muscle calcium channels. However, this subunit accelerates VDI of Ca_V_1.2 channels expressed in *Xenopus* oocytes or mammalian cells (Singer et al., 1991; Wei et al., 1991; Eberst et al., 1997).

**Figure 4:**
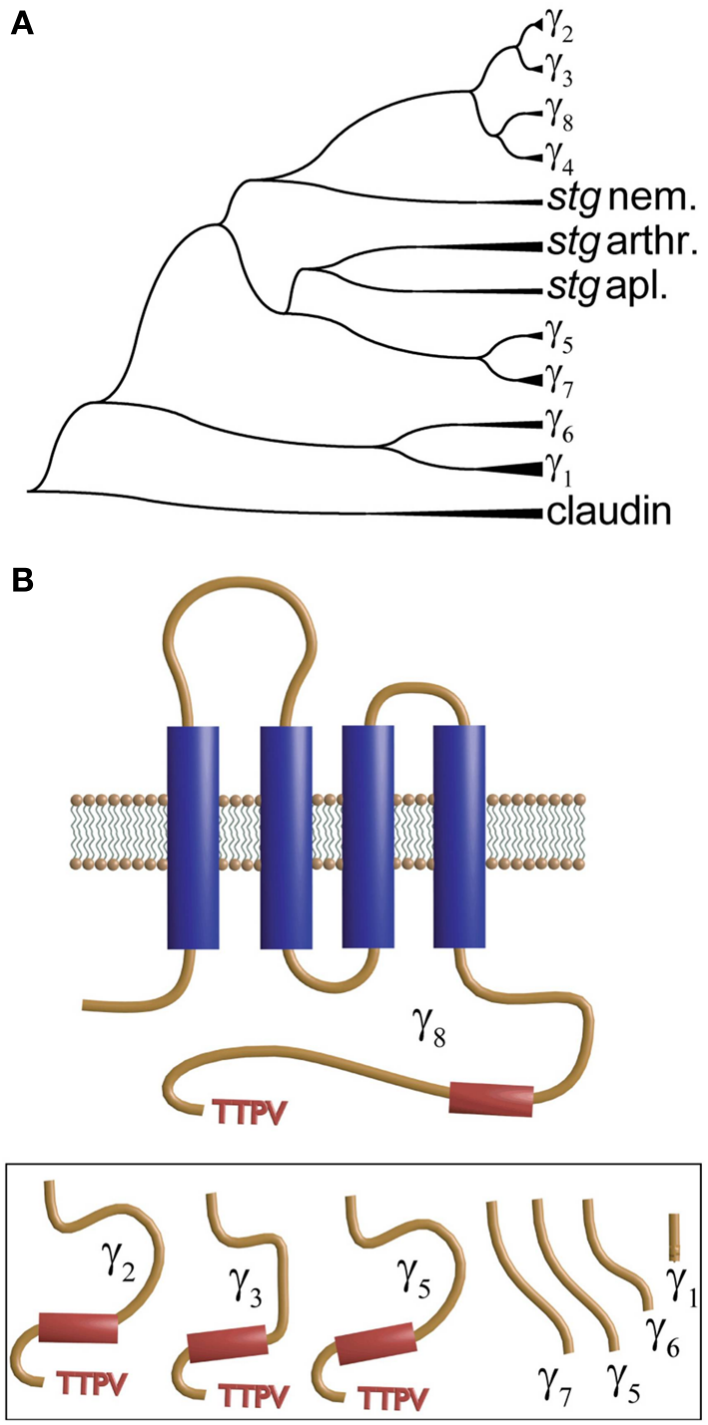
**Dendrogram and transmembrane topology of the γ subunit**. **(A)** Phylogenetic dendrogram constructed using CLUSTAL to align CACNG sequences from vertebrates (zebra fish, human and *Xenopus*) and invertebrates. Proteins labeled *stg* nem., *stg* arthr. and *stg* apl. refer to *stargazin* from nematode, arthropod and aplysia, respectively. **(B)** The γ-subunit encompasses four transmembrane domains and a C-terminus that varies significantly among the different forms and that in γ_2_, γ_3_, γ_5_ and γ_8_ (shown in the inset below) ends in a TTPV motif that tethers the protein to the PDZ domains.

The second member of the γ-subunit family was identified from mapping a mutation in a mutant mouse “s*targazer*” that displays ataxia and seizures. This mutation identifies a 38 kDa protein encompassing four membrane-spanning regions and a cytosolic N- and C-terminus that shared significant homology with γ_1_ and was thus named γ_2_ (Letts et al., 1998). This protein is also referred to as *stargazin* (Chen et al., 2000; Ferron et al., 2008). This new γ subunit produced a small but significant shift in the voltage dependence of the activation and inactivation of heterologously expressed neuronal calcium channels. However, what appeared to be more relevant to the phenotype was that granule cells from *stargazer* mice did not respond to the glutamatergic agonist alpha-amino-3-hydroxy-5-methylisoxazole-4-propionic acid (AMPA). More surprisingly, endogenous calcium currents recorded in granule cells lacking γ_2_ were indistinguishable from those recorded from wild type mice (Chen et al., 2000). This work also showed that γ_2_ co-immunoprecipitates with AMPA receptors and PSD-95, and the authors concluded that γ_2_ regulates delivery of AMPA receptors to post-synaptic membrane. The latter requires an intact C-terminus and interaction with PSD-95. This new role was the reason for naming these proteins TARPs for **t**ransmembrane **A**MPA receptor **r**egulatory **p**roteins (Coombs and Cull-Candy, 2009; Jackson and Nicoll, 2011). TARPS or γ subunits are members of the tetraspanning supergroup of membrane proteins that together with the tight junction component claudin give rise to the protein superfamily of claudin-like proteins and are represented by 8 different genes in the human genome. Figure 4A shows a phylogenetic tree of *stargazin* and γ subunits that was obtained by comparing a sequence of a variety of vertebrate and non-vertebrate species. Several human claudin sequences were also included. Each branch identified as γ-subunits (γ_1_–γ_7_) corresponds to vertebrate sequences while those from invertebrates are shown as *stargazin* since they diverge before the different vertebrate subunits. This analysis confirms the idea that all these proteins emerged from a single ancestral gene by tandem repeat and chromosome duplication (Burgess et al., 1999, 2001; Chen et al., 2007). An in-depth analysis of 24 mammalian γ-subunits revealed a similar grouping that clearly separates the skeletal muscle γ_1_ and γ_6_ isoforms from the rest (Chu et al., 2001). In all cases, including claudin and *stargazin* proteins, hydropathy analysis indicates the presence of four transmembrane segments that justify their name of tretraspanning proteins. Other shared features are that the loop joining the first and second transmembrane segment is the longest one, a conserved GLW of unknown significance is found in this loop, and that the intracellular loop joining the second and third transmembrane α-helix is the shortest. Although all contain N-linked glycosylation sites in the first extracellular loop, only in γ_1_ and γ_6_ are these sites localized before and after the signature GLW motif. The N- and C-termini seem to concentrate the most divergent features. Genes at the top branches of the phylogenetic tree code for subunits carrying a TTPV motif at the C-terminus. This sequence, required for binding to the PDZ motif of scaffolding proteins such as PSD-95, is missing in the lower branches of the phylogenetic tree (Figure 4B). This PDZ binding motif is critical for targeting AMPA receptors to the synaptic membrane and sets γ_2_, γ_3_, γ_4_, and γ_8_ apart as transmembrane AMPA receptor regulatory proteins or TARPs. The N-termini of muscle γ_1_ and γ_6_ lack a palmitoylation signal found in all other isoforms (Chen et al., 2007).

With respect to the functional role and interaction with the calcium channel, the best characterized case is γ_1_, the first γ-subunit cloned from skeletal muscle (Jay et al., 1990). When co-expressed in the oocyte with the cardiac Ca_V_1.2, it accelerates inactivation and shifts its voltage dependence toward more negative voltages (Jay et al., 1990; Eberst et al., 1997). This subunit is found exclusively in skeletal muscle (Eberst et al., 1997) and when removed by gene targeting there is an increase in calcium current density recorded on myotubes. In agreement with what was observed when co-expressed with Ca_V_1.2, inactivation was faster and started at more negative potentials in myotubes from wild type mice compared to γ^−/−^_1_ littermates (Freise et al., 2000). This phenotype predicts that γ_1_ decreases the amount of Ca^2+^ entering during an action potential, but it does not influence calcium release or excitation-contraction coupling (Ursu et al., 2001).

According to the most recent review by Hofmann et al. (2014), the calcium channel complex in the heart does not include a γ-subunit. However, three isoforms, γ_4_, γ_6_, and γ_7_, were originally pulled by RT-PCR from rat ventricle (Chu et al., 2001). This was later confirmed by experiments which in addition to identifying γ_8_ in human ventricles showed that all of them associated with heterologously expressed Ca_V_1.2 channels (Yang et al., 2011). Using HEK cells, the authors reported that co-expression of all four γ isoforms increased current density and shifted the voltage-dependence of activation toward more negative voltages to a variable degree. For the case of γ_8_, the left-shift occurred only in the presence of α_2_δ. What came as a surprise was that γ_6_ associates with Ca_V_3.1 channels in both cardiac and skeletal muscle (Hansen et al., 2004).

In the central nervous system, TARP γ-subunits associate with AMPA receptors with a variable stochiometry (Kato et al., 2010) and, moreover, calcium channels do not seem to be the main partner (Chen et al., 2000). What is the stoichiometry and the function of the γ-subunit in neuronal calcium channels remains to be answered. However, there is now strong evidence that γ_2_ (Sandoval et al., 2007) and γ_7_ (Ferron et al., 2008) regulate the forward traffic of the neuronal calcium channel. So it appears that the γ-subunits share with other accessory subunits the ability to modulate channel function as well as trafficking.

## Small GTPases

Small GTPases (also known as small GTP-binding proteins or the Ras superfamily of GTPases) are monomeric G proteins with low molecular masses ranging from 20 to 40 kDa (Wennerberg et al., 2005; Vigil et al., 2010; Cherfils and Zeghouf, 2013). They regulate diverse processes including gene expression and the GTP-bound form interacts with a multiplicity of cellular effectors. After activation, GTPase-activating proteins (GAPs) stimulate GTP hydrolysis and accelerate conversion to the inactivated GDP-bound state. Several members of this family share the ability to downregulate surface expression of HVA channels and some even stop expressed channels from opening, providing potent inhibitory mechanisms that work over different time scales (Yang and Colecraft, 2013).

**Figure 5:**
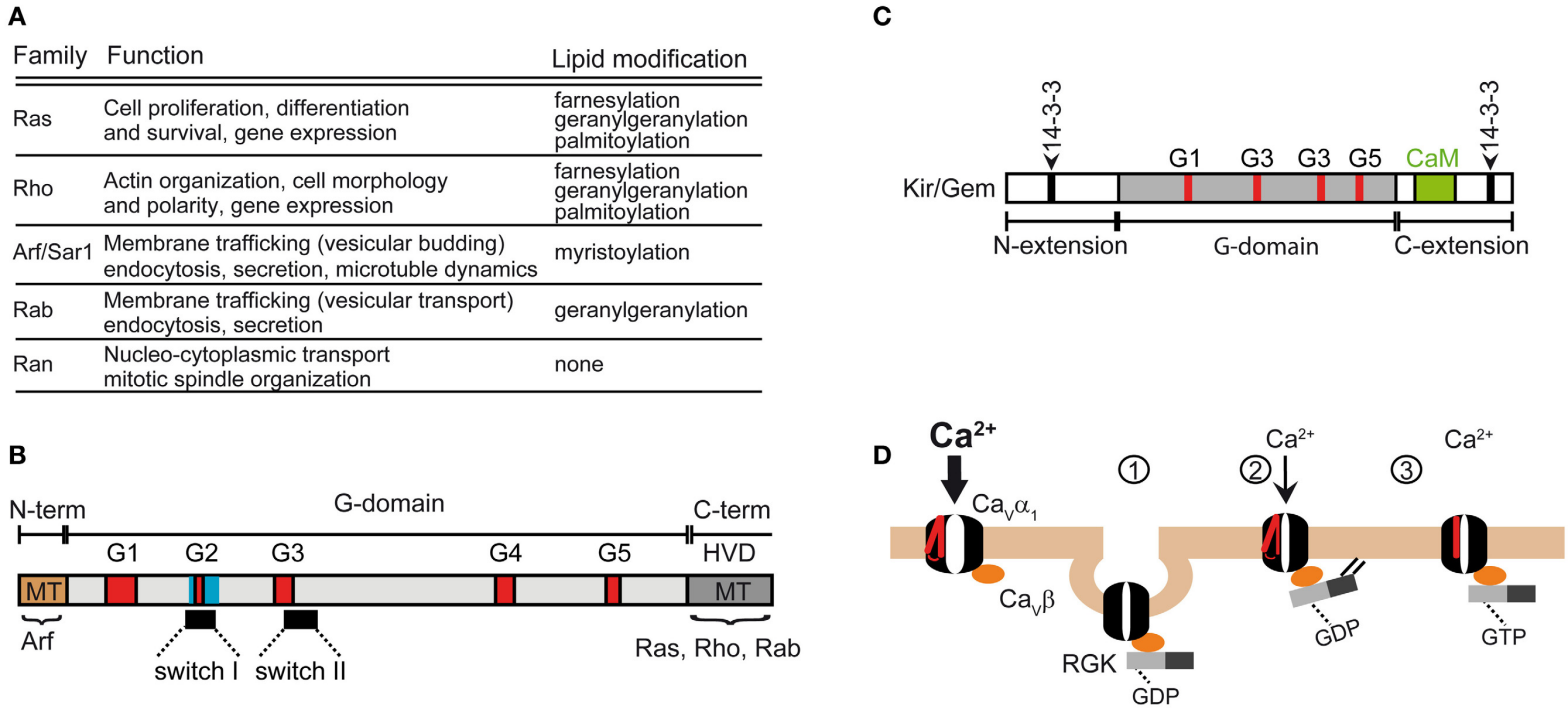
**Functions of the Ras superfamily of small GTPases**. **(A)** Subfamilies and functions of the Ras superfamily (reviewed in Vigil et al., 2010). **(B)** Domain structure shared by the Ras superfamily. The shared feature is the G-domain containing several GDP/GTP binding motifs (labeled G1 to G5). The Ras superfamily encompass a highly variable C-terminal domain (HVD) that includes the membrane targeting domain (MT) which undergoes lipid modification in Ras, Rho and Rab subfamilies. MT in Arf subfamily is at the N-terminus. **(C)** Domain organization of RGK GTPases. Note that the G domain differs from the canonical one shared by the other members of the Ras superfamily. Besides the Ca^2+^/calmodulin binding site (CaM), RGKs also contain two sites for 14-3-3 binding. The β-subunit of calcium channels binds to the G-box. **(D)** Models explaining RGK induced reduction of Ca^2+^ currents mediated by HVA channels (described in Yang et al., 2010). The normal function of HVAs requires the association of the β-subunit. Channel activation is preceded by the movement of the voltage sensor, represented in red. RGKs bound to GDP associate with the β-subunit and downregulate the channel by promoting its backward trafficking through a dynamin-dependent endocytosis (1) or by decreasing its open probability through a mechanism that relies on RGK membrane anchoring (2). Gating of the channel may be also altered by GTP-bound RGK through immobilization of the voltage sensor (3).

The superfamily of small GTPases is grouped into five main (canonical) families namely, Ras, Rho, Arf/Sar1 (ADP-rybosylation factor/secretion associated and Ras-related), Rab (Ras-related in brain), and Ran (Ras-related nuclear protein) that exhibit relatively separate cellular functions (Figure 5A). Except for members of the Ran family, small GTPases can undergo several lipid modifications that target them to specific cellular organelles, including ER, Golgi and small vesicles. The Ran family is found in the cytosol or nucleus where they regulate nucleocytoplasmic transport of RNAs and proteins. The Ras family regulates gene expression and the Rho members control actin-based cytoskeleton rearrangements, cell morphology and also gene expression. The Arf family participates mainly in the budding of vesicles while the Rab family is involved in vesicular transport. The latter have been widely used as markers due to their association with specific vesicle types.

The Ras superfamily shares a conserved core domain, the G domain, which contains five GDP/GTP binding motifs involved in binding GTP and phosphate/magnesium (Figure 5B; G1–G5) and two switch regions (I and II) that change conformation upon GTP-GDP cycle and determine the selective binding to regulatory proteins and to effectors via the core effector domain overlapping with switch I region. In addition to the G box, small GTPases contain N- and C-terminal extensions that are the target of post-translational modifications. The Ras, Rho, and Rab members contain a highly variable C-terminal region (HVD, Figure 5B) that undergoes lipid modifications facilitating specific membrane targeting (MT, Figure 5B) while the Arf subgroup encompasses an N-terminal extension that is myristoylated.

Besides the five classical families, the Ras superfamily includes additional related proteins, such as Ral, Rheb, Rit and RGK, whose classification into the five traditional groups is more or less subjective (Goitre et al., 2014). The RGK family is a relatively new protein subfamily that comprises four members, Rem, Rem2, Rad, Kir/Gem, which are thought to play an important role as regulators of voltage-activated calcium channels (for a review see Correll et al., 2008). Although RGK proteins have amino acid substitutions in conserved residues among Ras that are involved in nucleotide binding and hydrolysis, they do display GTPase activity (Splingard et al., 2007). Other distinguishing features of RGK are their relatively large N- and C-terminal extensions and the effector and switch regions (G-Box) which diverge from classical Ras and the fact that they do not undergo classical lipid modification (Opatowsky et al., 2006). The differences in the G-Box confer selectivity for intracellular effectors (Figure 5C). RGK protein contains binding sites for 14-3-3 protein, calmodulin and the calcium channel β-subunit.

The first work reporting that a small GTPase associates directly with a component of the HVA channel complex was published more than a decade ago (Beguin et al., 2001). Kir/Gem was shown to directly associate with the calcium channel β-subunit and decrease the channel's surface expression. The authors proposed a simple competition mechanism whereby RGK sequestrates the β-subunit preventing it from interacting with newly synthesized α_1_-subunits and impeding the release of the channel complex from the ER. This discovery added a new regulatory input to voltage-gated calcium channels that was later generalized for all RGKs (Beguin et al., 2001, 2005a,b, 2006; Finlin et al., 2003; Yang et al., 2010). This model originally assumed that the β-subunit binds either to the α_1_-subunit or to Kir/Gem. Later, the same authors and others proposed that Rem or Kir/Gem form a tertiary complex with the β subunit through the same domain (GK) that binds α_1_ (Finlin et al., 2006; Beguin et al., 2007). On the RGK side, the G-domain suffices for β-binding (Beguin et al., 2007).

Besides sequestration of the β-subunit to the cytoplasm, RGKs can also induce its nuclear retention by a mechanism involving calmodulin and 14-3-3 (Mahalakshmi et al., 2007a,b). Interestingly, a short β-subunit splice variant has been reported to localize in the nucleus and to bind to a chromatin binding protein resulting in gene silencing (Hibino et al., 2003). Thus, RGK can be important for translocating the β-subunit to the nucleus where it might directly affect gene expression in a GTPase-independent manner.

Regulation of voltage-activated calcium channels by small GTPases appears not to be limited to the surface expression of the channel but also applies to its gating. Overexpression of Rem 2 in HIT-T15 cells, a cell line derived from Syrian hamster islet cells resulted in calcium current inhibition that were not accompanied by a reduction in the number of L-type channels assembled in the plasma membrane as assessed by surface biotinylation (Finlin et al., 2005). Although the molecular basis for such a regulation is unknown, as suggested by the authors, it would provide a faster onset of the inhibition of calcium current density than that mediated by alterations in channel trafficking/expression. Likewise, overexpression of Rem 2 in rat sympathetic neurons virtually eliminated N-type currents (Chen et al., 2005). In heterologous expression systems (HEK 293 cells), a decrease in N-type calcium current density occurred without altering the channel surface expression determined by a live-cell radio-labeled ω-conotoxin, a specific N-type channel antagonist (Chen et al., 2005). The authors proposed a model in which binding of Rem 2 to pre-existing channels complexes containing the β-subunit leads to a non-conducting state by a yet to be discovered mechanism.

In an attempt to unify the variety of proposed mechanisms underlying the inhibition of the calcium channel function by RGK GTPases, Colecraft and colleagues studied the effects of Rem on Ca_V_1.2 heterologously expressed in HEK 293 cells and found that distinct conformations of the GTPase contribute to different, likely redundant, mechanisms for the reduction of L-type current (Yang et al., 2010) (Figure 5D). RGK proteins may decrease the number of channels expressed in the plasma membrane by increasing dynamin-mediated endocytosis of the channel complex. We previously demonstrated that the β-subunit associates directly with dynamin and promotes endocytosis (Gonzalez-Gutierrez et al., 2007). Together these results suggest that Rem 2-induced channel endocytosis is dependent on the β-subunit. The latter may recruit additional proteins to the endocytic sites. The β-subunit binds to dynamin through the SH3 domain suggesting that a complex including α_1_ and Rem and dynamin is plausible. In a primary culture of skeletal myoblasts, overexpression of Rem inhibits excitation-contraction coupling by decreasing the density of the functional L-type calcium channel without altering the ryanodine receptors or the calcium efflux from SR, but whether anterograde or retrograde trafficking is targeted has not been tested (Bannister et al., 2008).

Two other mechanisms of Rem action that might coexist with the stimulation of dynamin-mediated endocytosis would affect channel gating: a decrease in the open probability and immobilization of the voltage sensor (Yang et al., 2010). The GTP bound form would be responsible for charge immobilization (Figure 5D).

Although all RGK GTPases associate with the β-subunit it appears that regulation of calcium channel function does not rely on this association in all cases. Inhibition of Ca_V_2.1 (P/Q-type) channels by Gem GTPase does not require direct interaction with the β-subunit but with the Ca_V_2.1 subunit itself (Fan et al., 2010, 2012). It has also been shown that the reduction of the maximal gating charge moved during activation of Ca_V_1.2 channels mediated by Rem does not depend on binding of the β-subunit but on a direct association of Rem with the α_1_ pore-forming subunit (Yang et al., 2012). The proximal C-terminus of Ca_V_1.2 contains a Rem 2/Rad binding site that overlaps with the Ca^2+^/CaM interaction surface (Pang et al., 2010). Association of Rem with this site inhibits calcium current density and the effect is partially masked by CaM. Alterations in the kinetics of CDI by association of Rem with this site were observed suggesting an additional regulatory input for RGK regulation of channel activity. Whether the β-subunit is mandatory for the observed effect on calcium currents mediated by Rem/CT-terminus association has not been tested since all the experiments were performed in the presence of this subunit.

Although the vast majority of the studies of voltage-gated calcium channel regulation by small GTPases have been done for the RGK family (for an extensive review see Flynn et al., 2012), several examples have been extended to include other members of this superfamily. It has been reported that Rab11 regulates degradation of Ca_V_1.2 channels, constituting a novel mechanism for the regulation of calcium currents by small GTPase (Best et al., 2011). Lately, it has been proposed that Rap1 modulates neurotransmitter release in mouse primary cortical neurons by altering Ca_V_1.2 and Ca_V_1.3 function through an Erk1/2-mediated process (Subramanian et al., 2013). Whether alteration of L-type channels occurs via regulation of the channel gating or its surface expression remains to be elucidated.

The story of RGK modulation of calcium channels unfolded in the opposite direction to that of canonical auxiliary subunits. The latter started as regulators of function and then revealed their chaperone-like role. RGK protein came into play in the calcium channel field as a regulator of channel surface expression and only then revealed its ability to modulate channel gating.

## Calmodulin

L-type calcium channels have long been known to undergo a Ca^2+^-dependent inactivation (CDI) that is initiated by calcium entering through the channel (Eckert and Chad, 1984; Yue et al., 1990). An extensive search for the structural determinant of this process led to the discovery that calmodulin (CaM) tethered to the channel constitutes the calcium sensor for CDI (reviewed by Christel and Lee, 2012). CaM is one of the most abundant proteins in eukaryotes and is extremely well conserved: the three genes represented in the humans genome code for the same 148 amino acid sequence shared with 100% identity among vertebrates. Sequence identity goes down to 90% if all available sequences from animals and plants are compared (Kortvely and Gulya, 2004; Tidow and Nissen, 2013). This ubiquitous protein also has some surprises for the calcium channel field. The initial search for the structural determinant of the calcium-dependent inactivation (CDI) characteristic of L-type currents was sent off-track by two issues: (1) The identification of an EF-hand-like motif in the C-terminus of Ca_V_1.2, which was necessary for this phenomenon (de Leon et al., 1995; Peterson et al., 2000), and (2) the fact that the fast Ca^2+^ chelator BAPTA was unable to completely suppress it (Neely et al., 1994). However, point mutations of potential Ca^2+^-coordinating residues in the EF-hand motif had little effect on CDI (Zhou et al., 1997; Bernatchez et al., 1998). Following up on these findings and alerted by the fact that the canonical CaM binding sequence “IQ” was present at the C-terminus and necessary for CDI (Zuhlke and Reuter, 1998), Yue and co-workers designed a series of ingenious experiments to show that this ubiquitous calcium binding protein was tethered to the calcium channel and worked as the calcium sensor for CDI (Peterson et al., 1999; Erickson et al., 2001). Since eliminating endogenous CaM was out of the question, they proposed instead to engineer a mutant CaM that did not bind Ca^2+^. Co-expression of this mutated CaM with Ca_V_1.2 channels produced currents lacking CDI. They also showed that IQ peptides derived from Ca_V_1.2 bind to CaM-GST fusion proteins. Curiously, the IQ motif is highly conserved among HVA calcium channels thus giving rise to the question of why only L-type currents display robust CDI. One lead is that IQ sequences found in Ca_V_2.2 and Ca_V_2.3 bind CaM only at μM (Ca^2+^) and that CaM interaction with Ca_V_2.1-derived IQ was weak (Peterson et al., 1999; DeMaria et al., 2001). This is consistent with the fact that CDI in Ca_V_2.x is only manifested when buffering is kept to the bare minimum (0.5 mM EGTA) (Lee et al., 1999). More strikingly, if local calcium increases (5–10 mM EGTA) were constrained, currents underwent Ca^2+^-dependent facilitation (CDF) rather than CDI (Lee et al., 2000). In Ca_V_1.2 channels, CDF is observed under low calcium buffering conditions (0.5 mM EGTA) (Zuhlke et al., 1999, 2000). As discussed below, the key to this sign-reversal of Ca^2+^-CaM regulation of calcium channels is the relative orientation of CaM with respect to the IQ peptide.

**Figure 6:**
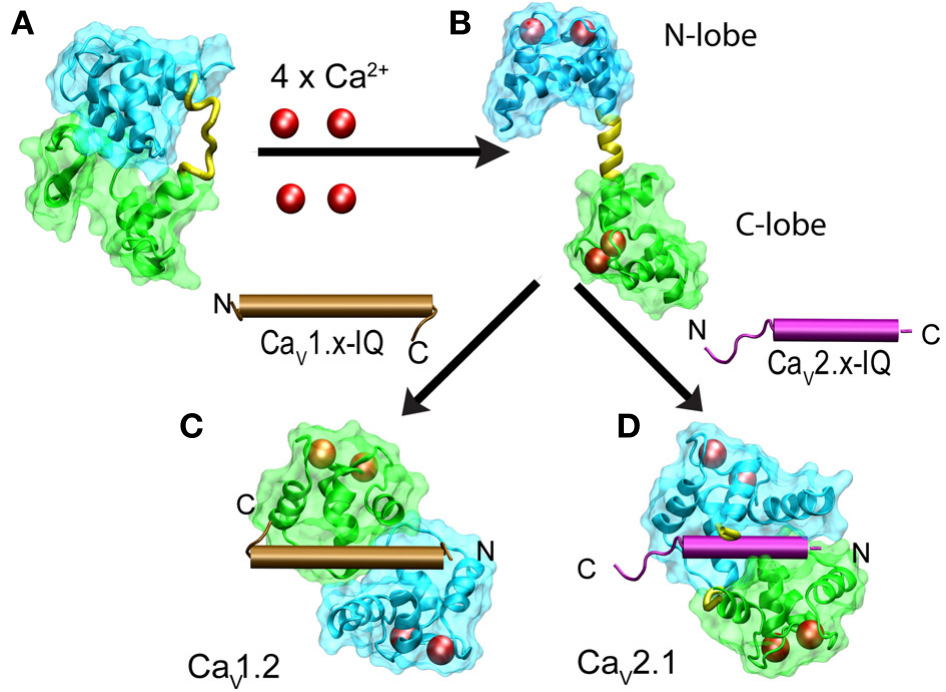
**Structural changes of calmodulin upon binding to Ca^2+^ and IQ in Ca_V_1.x and Ca_V_2.x channels**. The N- and C-lobes of CaM are shown in cyan and green, respectively while the linker is colored in yellow. Ca^2+^ ions are represented as red spheres and IQ as brown and violet cylinders for Ca_V_1.x and Ca_V_2.x, respectively. Binding of four Ca^+2^ to the dumbbell-like configuration of ApoCaM **(A)** promotes the extended conformation of CaM with an α-helical linker **(B)**. Binding of Ca^2+^-CaM to IQ brings the N- and C-lobes into close proximity. IQ from Ca_V_1.x binds to CaM in a parallel configuration **(C)** and IQ-Ca_V_2.x in an antiparallel manner **(D)**. PDB files are as follows: apoCaM: 1QX5, free Ca^2+^ CaM: 1CLL, CaM with Ca_V_1.2 IQ: 2BE6, CaM with Ca_V_2.2 IQ 3DVE, dimer CaM with Ca_V_1.2 IQ: 3G43.

CaM is a dumbbell-shaped protein with two homologous globular modules joined by a flexible linker (reviewed by Hoeflich and Ikura, 2002). Each of the modules carries two canonical EF-hand motifs that bind Ca^2+^ with different affinities. The C-terminus (C-lobe) is more sensitive to Ca^2+^that the N-terminus (N-lobe). Nuclear magnetic resonance studies revealed that in the absence of Ca^2+^, apoCaM has a dumbbell-like configuration with a linker lacking secondary structure and both lobes close to each other and thus with a reduced solvent exposure of hydrophobic residues (Kuboniwa et al., 1995; Zhang et al., 1995) (Figure 6A). Ca^2+^-CaM prefers an extended conformation with a relatively large solvent-exposed surface. In the presence of Ca^2+^, hydrophobic patches become exposed and the extended linker adopts an α-helical structure (Figure 6). Binding to any of the target peptides reduces the exposed surface by as much as 7000 Å^2^. In the vast majority of cases, the bound peptide runs antiparallel to CaM with the N-terminus closer to the C-lobe while the C-terminus contacts the N-lobe (Figure 6). There are also cases involving the formation of a dimer of dimers, in which two CaM retain their extended conformation and associate in an antiparallel orientation embracing two binding peptides which also run antiparallel. This is the configuration found in Ca^2+^-CaM in complex with the CaM binding peptide of the small-conductance calcium-activated potassium channel (Schumacher et al., 2004). Structures obtained with the IQ motif derived from different types of calcium channels revealed a full repertoire of configurations including a dimer of dimers and the parallel orientation found with Ca_V_1.2-derived peptides (Figure 6). Combining this information with the functional studies on how CDI and CDF were affected by selectively ablating Ca^2+^ binding to the high-affinity C-lobe or low-affinity N-lobe revealed a fascinating feedback mechanism that discriminates between the magnitude and spatial distribution of Ca^2+^. CDI in Ca_V_1.2 channels is resistant to Ca^2+^ buffering and requires an intact CaM C-lobe that is closer to the channel pore because CaM binds IQ in a parallel orientation. This leaves the N-lobe in a position where it can sample Ca^2+^ further away from the channel mouth to produce CDF in response to global changes in Ca^2+^. Experimentally, binding of Ca^2+^ to the N-lobe would only occur under low-buffering conditions. In Ca_V_2.2, CaM binds IQ in the canonical antiparallel orientation positioning the N-lobe close to the channel mouth. This gives rise to a CDF that is resistant to calcium buffering while a more distant C-lobe would promote CDI in a manner that is suppressed by mild calcium buffering.

Besides the IQ motif, other segments come into play such as that which seems to be responsible for the Ca^2+^-independent tethering of apoCaM to the channel. Initial studies showed that IQ can associate with CaM in the virtual absence of calcium. A mutant of CaM that does not bind calcium cannot associate with IQ. Since this CaM mutant prevented CDI it must associate with the channel to displace the endogenous CaM. A plausible explanation is that other regions of the channel, besides IQ, can bind apoCaM or the mutant CaM. Pitt et al. (2001) were able to identify a cytoplasmic segment of Ca_V_1.2 that binds the mutant CaM, which they termed the Pre-IQ motif. This motif is upstream and partially overlaps with the IQ sequence suggesting that at extremely low Ca^2+^ CaM binding may be shifted from the IQ to the Pre-IQ and thus the structure of the Ca_V_α_1_/CaM complex for the mutant and wild-type CaM may differ.

To add to this already complex picture, Yue and coworkers identified an additional CaM binding site that influences CDI on Ca_V_1.2 and Ca_V_1.3 channels. This new binding site, referred to as NSCaTE, which stands for **N**-terminal **S**patial **Ca**^2+^ **T**ransforming **E**lement, is located at the very proximal N-terminus of Ca_V_1.2 and Ca_V_1.3 channels (Dick et al., 2008). By binding to this motif, the N-lobe of CaM switches from a global to a local Ca^2+^ sensor. Introducing NSCaTE into Ca_V_2.2 which lacks this element endows these channels with a CDI that is resistant to high Ca^2+^ buffering. The NSCaTE motif present in a large number of invertebrate homologs of Ca_V_1.x is followed by a second methionine suggesting that cells can fine tune this CDI by alternating the initiation point of translation (Taiakina et al., 2013). In neurons, the calcium binding protein CaBP1 also participates in CDI regulation of Ca_V_1.x by competing with CaM for the IQ domain. When the channel is bound to CaBP1, CDI is eliminated and sustained calcium influx follows (Findeisen and Minor, 2010; Findeisen et al., 2013).

Specific cells and channels seem to have developed additional mechanisms to regulate the sensitivity to calcium. In rodent cochlear inner cells, for example, Ca_V_1.3 channels that mediate fast voltage-dependent calcium influx change their sensitivity to internal calcium during animal development. In gerbil, as the animal matures and starts to hear, CDI is stronger in inner cells at the base of the cochlea where cells sense high frequencies (Johnson and Marcotti, 2008). In mouse, mature inner hair cells, Ca_V_1.3 channels also exhibit greater CDI than in immature cells and behave in a manner more similar to what is observed in heterologous expression systems in which CDI is resistant to high Ca^2+^ buffering. In contrast to inner cells, there is a significant reduction in CDI when Ca^+2^ buffering is increased. Thus, as the cell matures CDI switches from a global sensing to a local sensing mode (Inagaki and Lee, 2013). It is tempting to link this developmental switch to the RNA editing of the IQ domain as recently reported for Ca_V_1.3 in some neurons (Huang et al., 2012). This exquisite flexibility of CDI found in Ca_V_1.3 channels was recently reviewed extensively by Ben et al. (2013). These recent findings also illustrate that Ca_V_α_1_/CaM interaction appears to be far more dynamic than expected from a stable stoichiometric component as it has been proposed and involves several alternative configurations subject to regulation.

## Concluding remarks

**Figure 7:**
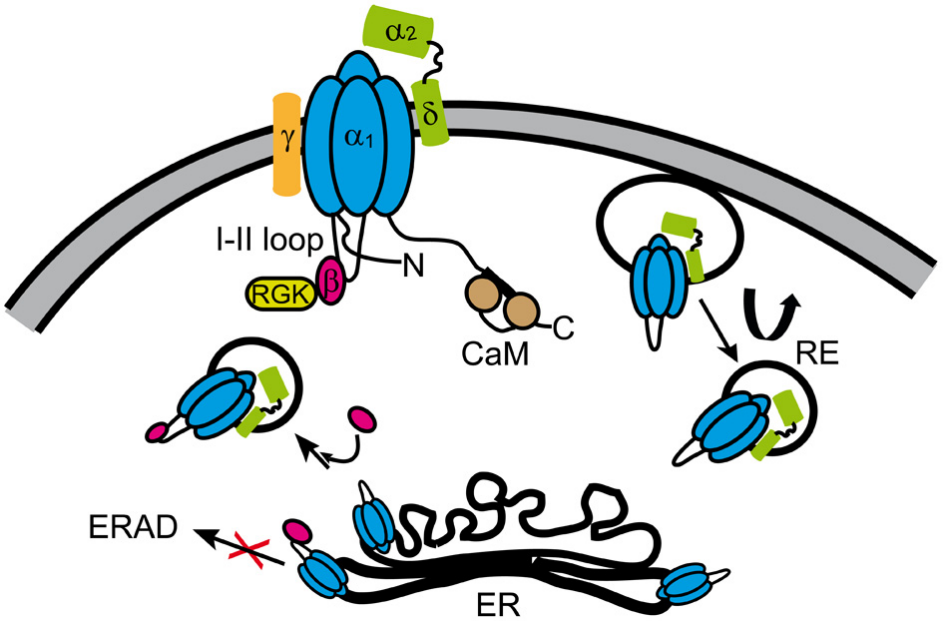
**Summary of proteins interacting with Ca_V_α_1_ of HVA channels**. HVA channels are heteromultimers consisting of at least three subunits (α_1_, β, α_2_δ), except for Ca_V_1.1 class that also includes the γ-subunit. For clarity, the α_1_-subunit sketch includes only the intracellular loop joining the first and second repeat (loop I–II) that associates with the β-subunit. The α_2_δ- and β-subunits increase the electrical activity and the number of the channels in the plasma membrane. While the β-subunit enhances anterograde trafficking of channels likely retained in the ER, α_2_δ-subunit promotes recycling of channels (curved arrow). The β-subunit also prevents the channel to be targeted to the ERAD degradation complex from the ER. RGKs regulate channel function by multiple mechanisms upon binding to the channel complex through the β-subunit (see Figure 5). CaM is widely known for its effect on HVA calcium dependent inactivation and facilitation. The exact subunit composition of the channel in the different vesicular compartments is not known. CaM, calmodulin; RGK, RGK small GTPases; RE, recycling endosome; ER, endoplasmic reticulum; ERAD, endoplasmic reticulum-associated protein degradation complex.

In this review, we focused on protein-protein interactions with the central pore-forming subunits of HVA calcium channels that are thought to participate in the formation of channel complexes underlying L-, N-, P/Q-, and R-type currents (Figure 7). It is not a coincidence that most of the α_1_-interacting proteins regulate the electrical activity as well as trafficking synergistically. The same protein is endowed with two separate effectors to either up regulate or down regulate calcium currents. β-subunits facilitate channel opening and promote forward trafficking while RGKs favor endocytosis and prevent channels from opening.

Calcium channels are probably one of the most highly regulated ion channel family because the calcium ion is an ubiquitous intracellular second messenger and thus calcium influx must be spatially and temporally finely tuned according to the requirement of the effector system. Besides coordinating the plethora of Ca^2+^ signal cascades, the cell must prevent toxic calcium overload that can lead to apoptosis. Calcium channels use an extensive repertoire of regulatory mechanisms to achieve tight regulation of the Ca^2+^influx. In addition to the canonical allosteric regulation by protein ligands, it becomes more evident that dynamic adjustment of their protein binding partners constitutes a sophisticated mechanism to further regulate calcium influx. A nice example of this is the finding that the β-subunit not bound to the α_1_-subunit may engage in dynamin-dependent endocytosis and stimulate internalization of membrane complexes including Ca_V_α_1_ (Gonzalez-Gutierrez et al., 2007).

In native systems, calcium channels are part of supramolecular assemblies containing effectors, regulatory proteins and elements responsible for feedback control. This assembled “factory” is then able to activate the right signaling pathway within a short time. Choosing the right partners for this assembly proves to be a relevant mechanism for regulating calcium signal at different time scales. For example, in heart and brain Ca_V_1.2 is co-assembled with β_2_ adrenergic receptor, adenylyl cyclase, kinase and kinase anchoring protein (AKAP) (Davare et al., 2001; Harvey and Hell, 2013). Phosphorylation of Ca_V_1.2 channels within the assembly produces a long-lasting increase in the probability of being open, thus augmenting the strength of the calcium signal.

The existence of these supramolecular complexes suggests the idea that trafficking and assembly machineries function in a concerted way to target the right components of the “factory” to specific sites in the plasma membrane. Compartmentalization of calcium channels within the cell surface appears to be another plausible mechanism to coordinate calcium signaling. For instance, in the heart it has been proposed that Ca_V_1.2 fulfilling different functions is spatially segregated. Ca_V_1.2 located in the T-tubules initiates calcium-induced calcium release while those destined for the caveolae regulate gene transcription (Shaw and Colecraft, 2013).

In summary, calcium channel complexes emerge as a rather dynamic system whose subunit composition varies. The question of what is the stoichiometry of HVA calcium channel then appears to be no longer valid. Perhaps the most loyal and specialized partner of HVA channels is the α_2_δ-subunit, while the β-subunit shares other destinations besides the α_1_ subunit (Hidalgo and Neely, 2007). With respect to γ-subunits, only γ_1_ proves to be a faithful partner of the skeletal muscle Ca_V_1.1 while the jury is still out on the rest of the γ-and α_1_-subunits which may even extend to Ca_V_3.x as possible candidates. CaM is a ubiquitous protein that only recently emerged as a stable partner of HVA channels but it follows flexible engagement rules.

### Conflict of interest statement

The authors declare that the research was conducted in the absence of any commercial or financial relationships that could be construed as a potential conflict of interest.
